# Inferring Nonlinear Gene Regulatory Networks from Gene Expression Data Based on Distance Correlation

**DOI:** 10.1371/journal.pone.0087446

**Published:** 2014-02-14

**Authors:** Xiaobo Guo, Ye Zhang, Wenhao Hu, Haizhu Tan, Xueqin Wang

**Affiliations:** 1 Department of Statistical Science, School of Mathematics & Computational Science, Sun Yat-Sen University, Guangzhou, China; 2 Southern China Research Center of Statistical Science, Sun Yat-Sen University, Guangzhou, China; 3 Zhongshan School of Medicine, Sun Yat-Sen University, Guangzhou, China; 4 Department of Physics and Informatics, Shantou University Medical College, Shantou, China; 5 State Key Laboratory of Ophthalmology, Zhongshan Ophthalmic Center, Sun Yat-Sen University, Guangzhou, China; Leibniz-Institute for Farm Animal Biology (FBN), Germany

## Abstract

Nonlinear dependence is general in regulation mechanism of gene regulatory networks (GRNs). It is vital to properly measure or test nonlinear dependence from real data for reconstructing GRNs and understanding the complex regulatory mechanisms within the cellular system. A recently developed measurement called the distance correlation (DC) has been shown powerful and computationally effective in nonlinear dependence for many situations. In this work, we incorporate the DC into inferring GRNs from the gene expression data without any underling distribution assumptions. We propose three DC-based GRNs inference algorithms: CLR-DC, MRNET-DC and REL-DC, and then compare them with the mutual information (MI)-based algorithms by analyzing two simulated data: benchmark GRNs from the DREAM challenge and GRNs generated by SynTReN network generator, and an experimentally determined SOS DNA repair network in *Escherichia coli*. According to both the receiver operator characteristic (ROC) curve and the precision-recall (PR) curve, our proposed algorithms significantly outperform the MI-based algorithms in GRNs inference.

## Introduction

With the development of high throughout technologies, gene expression data has provided an excellent approach to investigate the underlying regulatory mechanism of cellular machines [1]. Inferring gene regulatory networks (GRNs), which explicitly depicts the regulatory processes, from expression data is still one of the most important topics in system biology [2] nowadays. But it is still a challenging problem due to the combinatorial nature of the problem and the poor information content of the data [3]. Therefore, developing powerful and computationally effective methods is critical for GRNs inference. To this end, the DREAM (Dialogue for Reverse Engineering Assessments and Methods) program and its conference series are devoted to encourage researchers to investigate novel powerful methods [4]-[6].

It is extremely important to detect nonlinear dependence in GRNs inference because the nonlinear regulatory relationship is common in biology [7]. Among numerous measurements of nonlinear dependence, mutual information (MI) has often been applied in modeling the dependence between genes since it is a natural generalization of correlation and can characterize the nonlinear dependence [7], [8]. A series of MI-based methods have succeeded in inferring the GRNs such as ARACNE [9], CLR [10], REL [11], MRNET [12] and PCA-MI [13]. Even though the MI is quite popular, it still has its limits. For example, to evaluate the MI usually involves the probability or density estimator which is challenging especially for multivariate variables. When the variables are continuous, the MI estimation is not so easy and the commonly used strategy is to discretize the data [14] and then to estimate the MI based on these discretized data, e.g., empirical estimator [15], Miller-Madow [15], shrinkage [16] and the Schurmann-Grassberger mutual information estimator [17]. PCA-MI [13] is another example which requires the assumption of normal distribution. But, it is not realistic because the gene expression data may strongly deviate from normality [18].

Recently, a novel measurement of dependence, distance correlation (DC) [19], has emerged as an elegant tool in evaluating nonlinear dependence, thanks to its appealing features. The DC has proven its power and computational effectiveness in detecting nonlinear dependence for two variables with arbitrary dimensions [19]-[21].Unlike the MI estimator, the DC estimation is quite simple without any distribution assumption. Surprisedly, to our best knowledge, the DC has seldom attracted the attention of the bioinformatics community.

In this article, we intend to incorporate the DC into GRNs inferring algorithms and validate the performance of DC-based GRNs inferring algorithms. Unlike traditional approaches, we employ the DC to represent the dependence between a pair of genes. This modification is simple yet critical due to the power and computational effectiveness of the DC. The results of two simulated data and real data suggest that the DC-based approaches can improve the accuracy and sensitivity of the GRNs inference.

## Methods

In this section, we will review the definitions of the MI and the DC, and then incorporate the DC into GRNs inference.

### Mutual Information

The MI of discrete random variables 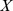 and 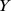 is defined as
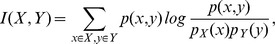
where 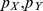 are the marginal probability mass functions of 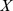 and 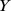, respectively, and 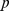 is the joint probability mass function of 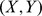.

For continuous random variables, the MI is defined as
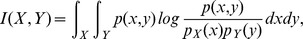
where 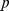 is the joint probability density function of 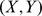 and 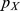 and 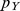 are the probability density functions of 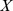 and 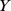, respectively.

In order to calculate the MI, it is necessary to first estimate the unknown probability density or mass functions 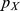, 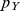 and 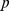.

### Distance Correlation

Distance correlation proposed by [19] is a creative way to detect the dependence. The key idea is to measure the discrepancy between the joint characteristic function and the product of its marginal characteristic functions in a special weighted 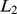 space. Specifically, for random variables 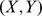, denote the joint characteristic function of 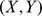 by 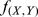, and its marginal characteristic functions 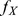 and 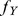. The distance covariance between 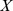 and 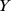 is defined as the root of the following equation:
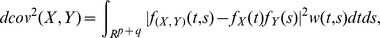
where 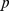 and *q* are the dimensions of 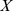 and 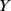, respectively, and 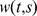 is the weight function given by 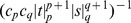 with 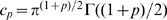 and 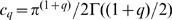. By standardizing the distance covariance, the distance correlation can be defined as,



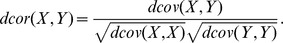



It can be shown that the empirical distance covariance for a given iid sample 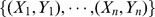 can be calculated by
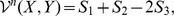
where



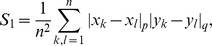





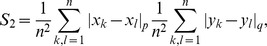





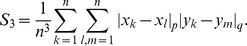



The empirical form of DC is quite simple in terms of the norms and does not involve the probability density estimator like the MI.

### DC-based GRNs Inference

A central role in GRNs inference algorithms is the dependence matrix 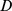, whose 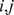 element 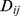 measures the dependence between variables (genes) 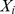 and 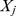. In nonlinear GRNs inference algorithms, the MI is the common choice in characterizing the nonlinear association between genes, that is
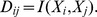



However, as discussion above, estimating mutual information is a tough task and the estimator is usually biased and unstable [12], [14]. Here, we use the DC as an alternative measurement to model the dependence matrix, that is
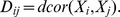



To verify the importance of incorporating the DC into inferring GRNs, we consider three popular gene regulatory network inference algorithms, CLR, MRNET and REL [10]-[12], and compare the performances of DC-based algorithms and MI-based algorithms. For the sake of clarification, we denote the MI-based algorithms by CLR-MI, MRNET-MI and REL-MI and the DC-based algorithms CLR-DC, MRNET-DC and REL-DC, repectively.

## Results

In this section, we present the results of different methods based on simulated data and real data.

### Validation

The performance will be evaluated by receiver operator characteristic (ROC) curve and precision-recall (PR) curve. The ROC curve is a graphical tool in evaluating the predictive results in order to avoid choosing the threshold. However, the ROC curve may overestimate the performance of the GRNs inference method due to the sparsity of GRNs [22]. The PR curve is recommended to be an alternative to the ROC curves [23]. Here we use ROC curve, which is a scoring metric adopted by DREAM3, as well as PR curves to evaluate the methods. The areas under ROC curve (AUC) and PR curve are also calculated.

### Evaluation on Simulation Data

#### Simulated data from DREAM challenge

We first evaluate our methods based on the widely-used Yeast knock-out gene expression data with size 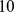, 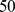, and 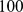 from DREAM3 in-silico network challenge [4]–[6]. DREAM challenge is a dialogue for Reverse Engineering Assessments and Methods, which provides a standard assessment of GRNs inference methods. In the DREAM3 challenge, the Yeast knock-out gene expression data and their gold standard networks are given.

**Figure 1:**
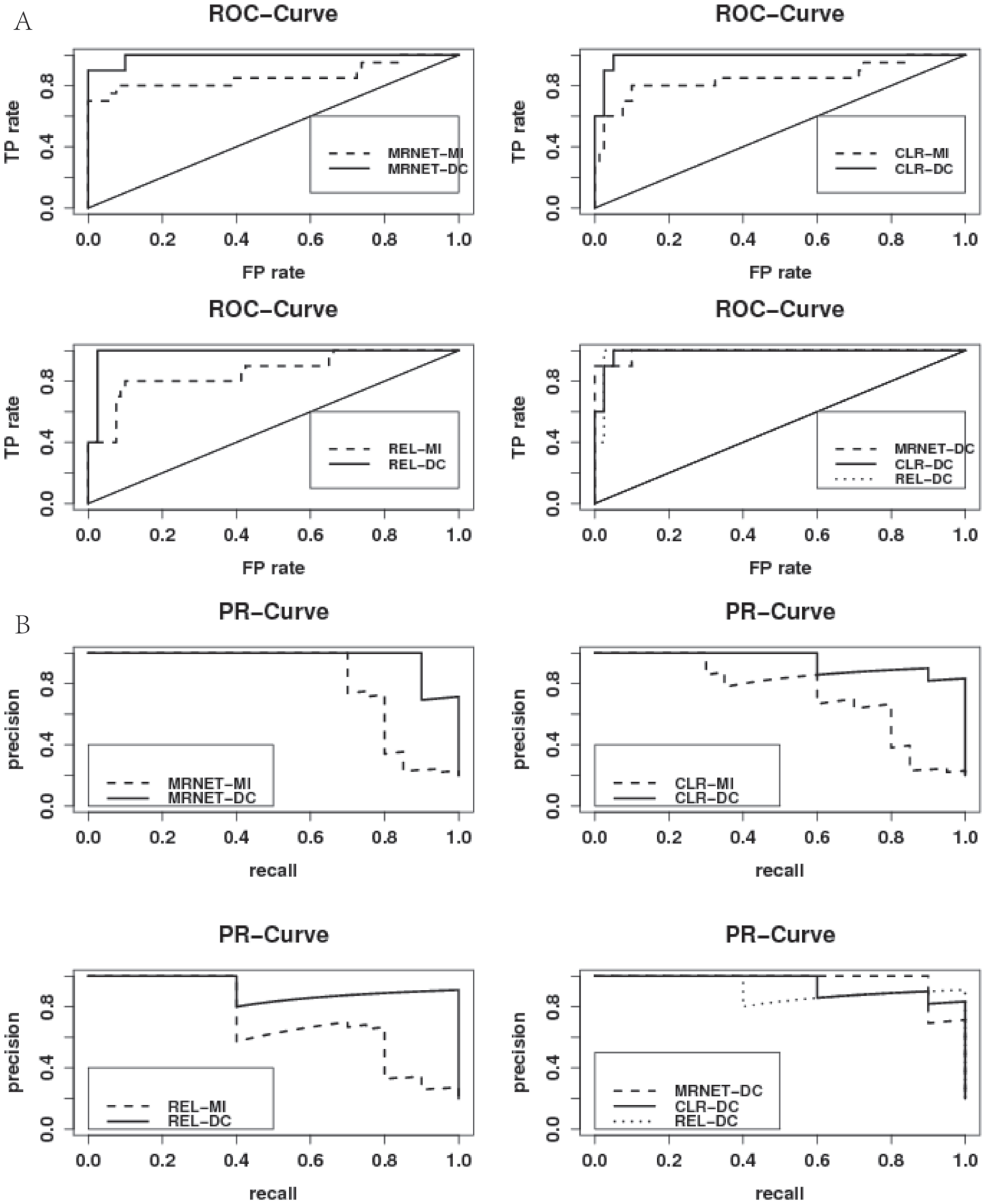
The performance of different methods on DREAM3 challenge Yeast dataset in size 10. (A) The ROC curves of different methods. (B) The PR curves of different methods. TP rate = TP/(TP+FN), FP rate = FP/(TP+TN), precision = TP/(TP+FP), recall = TP/(TP+FN), where TP, FP, TN and FN are the numbers of true positives, false positives, true negatives and false positives, respectively.

In order to clearly compare the performance of different methods, the ROC curves and the PR curves are plotted in Figure 1. Figure 1A presents the ROC curves on DREAM3 challenge Yeast dataset with size 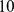. Figure 1A shows that the DC-based algorithms perform much better than the corresponding MI-based algorithms, the DC therefore has high power in characterizing the nonlinear regulatory relationship. We can observe the similar results of the PR curves in Figure 1B. The ROC curves and the PR curves of three different algorithms on Yeast gene expression data with size 50 and 100 are described in Figure S1–S2. In these two cases, the DC-based algorithms consistently outperform the MI-based algorithms.

**Figure 2:**
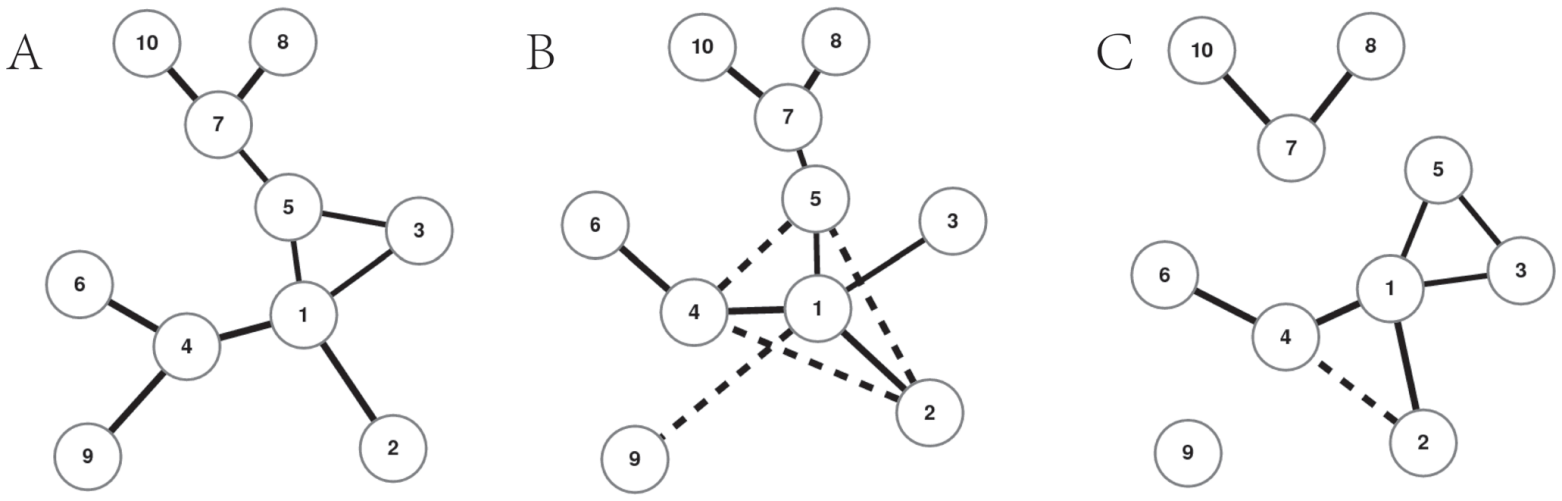
Networks inferred from DREAM3 challenge Yeast dataset with size 10. (A) The true network with 10 nodes and 10 edges. (B) Network Inferred by using the REL-MI method. The dashed lines G5-G4,G5-G2,G4-G2 and G1-G9 are false positives, while G4-G9 and G3-G5 are false negative. (C) Network Inferred by using the REL-DC method. The dashed lines G4-G2 is false positives, while G4-G9 and G5-G7 are false negative.

Figure 2 displays the networks inferred by using the REL-MI and REL-DC methods based on the DREAM3 challenge Yeast dataset with size 10. To equally comparing the performance of the REL-MI and REL-DC methods, we set the true positive rates of both methods to be 0.8 and then compare the false positives. We can observe from Figure 2B that there are four false positives G5-G4,G5-G2,G4-G2 and G1-G9 by REL-MI. The four non-existing regulations G5-G4,G5-G2,G4-G2 and G1-G9 are probably incurred by the co-regulators, while the MI-based methods work on the pair-wise association only. Figure 2C displays the network inferred by using the REL-DC method. Obviously, the false positives G5-G4,G5-G2 and G1-G9 are successfully removed by REL-DC, which indicates that the DC-based methods work well in distinguishing direct (or causal) interactions from indirect associations.

Table 1 provides the results for three different methods on DREAM3 challenge Yeast dataset with size 10, 50,and 100, respectively. The results indicate that the DC-based methods improve greatly the accuracy of GRNs inference compared with the MI-based methods.

**Table 1:** The ROC areas and the PR areas of different methods on DREAM3 challenge Yeast dataset with size 10, 50, 100 and Syndata, respectively.

Method	CLR-MI	CLR-DC	MRNET-MI	MRNET-DC	REL-MI	REL-DC
ROC area						
Size10	0.83	0.99	0.81	0.99	0.86	0.99
Size50	0.79	0.89	0.76	0.89	0.79	0.89
Size100	0.8	0.87	0.78	0.85	0.75	0.86
PR area						
Size10	0.63	0.94	0.5	0.97	0.72	0.92
Size50	0.19	0.5	0.16	0.52	0.34	0.47
Size100	0.15	0.43	0.14	0.36	0.12	0.35

#### Simulated data from SynTReN

In this section, we also compare the performance of different methods in another simulated datasets generated by SynTReN network generator [24]. SynTReN network generator is used to create synthetic transcriptional regulatory network and respective simulated data from the source networks with different levels of noise. Here, the synthetic transcriptional regulatory networks are generated from *Escherichia coli* and the number of nodes is set to be 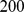, in which there are 100 nodes in background network. We can observe from Table 2 that the DC-based algorithms consistently outperform the corresponding MI-based algorithms. For simplicity, the ROC and PR curves of different methods with different levels of noise are deferred to Figure S3–S5. Figure S3–S5 also demonstrate that the DC-based algorithms are superior to the MI-based algorithms in characterizing non-linear dependence.

**Table 2:** The ROC areas and the PR areas of different methods on SynTReN datasets with noise 0.1, 0.2, 0.3, respectively.

Method	CLR-MI	CLR-DC	MRNET-MI	MRNET-DC	MI	DC
ROC area						
0.1 noise	0.78	0.86	0.78	0.84	0.75	0.84
0.2 noise	0.63	0.73	0.63	0.72	0.57	0.64
0.3 noise	0.63	0.72	0.62	0.73	0.57	0.64
PR area						
0.1 noise	0.09	0.20	0.06	0.11	0.06	0.07
0.2 noise	0.07	0.14	0.06	0.09	0.06	0.07
0.3 noise	0.07	0.14	0.06	0.10	0.07	0.09

### Evaluation on Real Gene Expression Data

**Figure 3:**
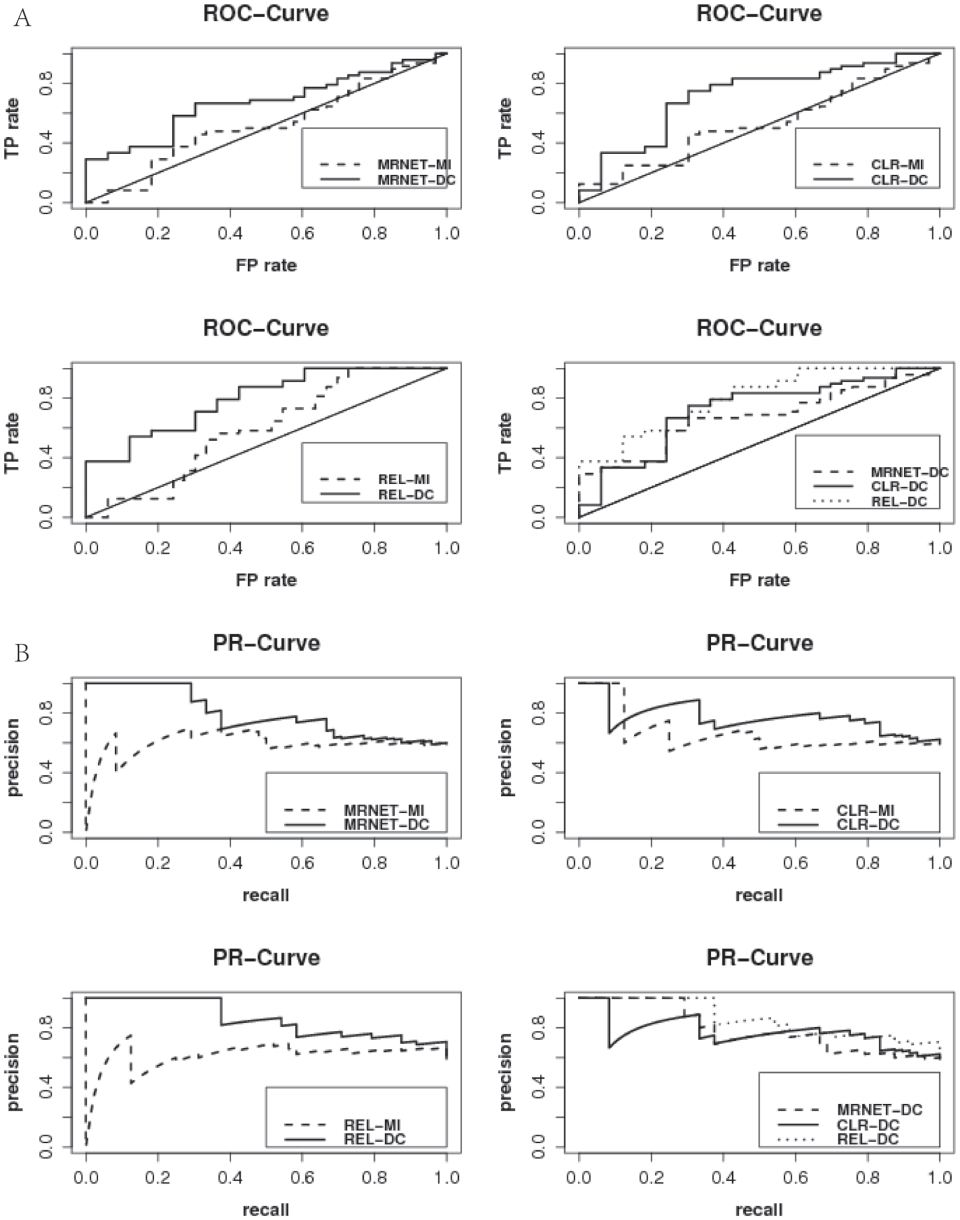
The performance of different methods on *Escherichia coli*. (A) The ROC curves of different methods. (B) The PR curves of different methods. TP rate = TP/(TP+FN), FP rate = FP/(TP+TN), precision = TP/(TP+FP), recall = TP/(TP+FN), where TP, FP, TN and FN are the numbers of true positives, false positives, true negatives and false positives, respectively.

We investigate the performance of the DC-based methods in the well-known SOS DNA repair network and experiment dataset in *Escherichia coli* [25]. Figure 3 presents the ROC and PR curves for the MI-based algorithms and the DC-based algorithms, and Table 3 presents the ROC and PR areas for different methods. All of these compared results demonstrate the superiority of the DC-based methods on this real gene expression data.

**Table 3:** The ROC areas and the PR areas of different methods on SOS network in E.coli.

Method	CLR-MI	CLR-DC	MRNET-MI	MRNET-DC	REL-MI	REL-DC
ROC area						
SOS	0.53	0.72	0.52	0.78	0.59	0.85
PR area						
SOS	0.66	0.77	0.59	0.79	0.61	0.86

## Discussion

In this article, we integrate the recently developed DC into GRNs inference algorithms and verify the power and computational effectiveness of the DC in inferring GRNs through three well-known GRNs inference algorithms: CLR, MRNET and REL. After comparing them carefully with the existing MI-based algorithms, we find that the proposed DC-based algorithms can uncover the nonlinear dependence more powerfully, and increase the accuracy in inferring GRNs. Although we only incorporate the DC into three MI-based GRNs inference algorithms, our idea can be extended to any other MI-based algorithms.

The DC has several strengths in comparison with the MI. Firstly, both the DC and the MI are nonparametric and have the property that the DC or the MI of two random variables equals to zero almost surely if and only if these two variables are independent, but the MI estimators need to discretize the variables which may not utilize the data sufficiently and lower the power. Secondly, the DC has computational effectiveness in detecting nonlinear dependence between multivariate variables [20]. Lastly but importantly, the DC can directly investigate the joint regulations of at least two sets of target genes. However, to the best of our knowledge, MI can work on pair-wise regulations in GRNs well. Despite the three-way mutual information (MI3) [26], a modified version of MI, has been designed to detect the co-regulators of target genes, the extension is tricky and MI3 can only detect two of the co-regulators [13].

We also compare the DC with another recently developed dependence measurement, maximum information criterion (MIC) [27] in GRNs inference. Table S1, S2 and S3 display the results of the MIC-based algorithms and the DC-based algorithms. The results suggest that the DC-based algorithms still outperform the MIC-based ones significantly in inferring GRNs.

However, none of the MI-based, the MIC-based or the DC-based methods is capable of detecting edge directionality. This issue can be partially alleviated by a two-stage procedure: the pair-wise association is detected first, and then the edge directionality is inferred using some specified methods such as linear regression [28]. In any case, this issue is not well understood. Furthermore, the proposed DC-based algorithms are designed to detect the direct interaction. Extending the DC-based methods to distinguish the direct interactions from indirect ones can help identify the false positive interactions hence increasing the detecting power [13]. Interestingly, comparing with the MI-based methods, the DC-based methods perform much better in distinguishing direct or causal interactions from indirect associations even though the DC considers the unconditional correlation only.

## Conclusion

In this paper, we introduce the DC-based algorithms for GRNs inference. The DC has appealing features such as computational effectiveness, no normality assumption and high power in detecting nonlinear dependence. Both of the simulated data and the real data analysis show that the proposed DC-based algorithms performs better than the corresponding MI-based algorithms. In conclusion, the DC-based methods can be served as a starting-point to characterize complex regulation relationship between genes, but not limit to infer GRNs.

## Supporting Information

Figure S1 **Comparison of the performance of different methods on DREAM3 challenge Yeast dataset in size 50. (A) The ROC curves of different methods.(B) The PR curves of different methods.** The plots show that the DC-based methods perform consistently much better than the MI-based methods, which demonstrate the superiority of DC in detecting non-linear regulatory relationship between genes.
(EPS)

Figure S2 **Comparison of the performance of different methods on DREAM3 challenge Yeast dataset in size 100. (A) The ROC curves of different methods.(B) The PR curves of different methods.** The plots show that the DC-based methods perform consistently much better than the MI-based methods, which demonstrate the superiority of DC in detecting non-linear regulatory relationship between genes.
(EPS)

Figure S3 **Comparison of the performance of different methods on SynTReN dataset with 0.1 noise. The number of nodes in the networks was 200. (A) The ROC curves of different methods. (B) The PR curves of different methods.** The plots show that DC-based methods perform consistently better than MI-based methods, even though the difference are not obvious in some cases.
(EPS)

Figure S4 **Comparison of the performance of different methods on SynTReN dataset with 0.2 noise. The number of nodes in the networks was 200. (A) The ROC curves of different methods. (B) The PR curves of different methods.** The plots show that DC-based methods perform consistently better than MI-based methods, even though the difference are not obvious in some cases.
(EPS)

Figure S5 **Comparison of the performance of different methods on SynTReN dataset with 0.3 noise. The number of nodes in the networks was 200. (A) The ROC curves of different methods.(B) The PR curves of different methods.** The plots show that DC-based methods perform consistently better than MI-based methods, even though the difference are not obvious in some cases.
(EPS)

Table S1 **Comparison of ROC area and PR area of MIC-based algorithms and DC-based algorithms on DREAM3 challenge Yeast dataset in size 10, 50, 100, respectively.** All of the results show that DC is significantly superior to the MIC in GRNs inference, which demonstrate that the DC is a powerful dependence measure in inferring GRNs.
(DOCX)

Table S2 **Comparison of ROC area and PR area of MIC-based algorithms and DC-based algorithms on SynTReN datasets with noise 0.1, 0.2, 0.3, respectively.** All of the results show that DC is significantly superior to the MIC in GRNs inference, which demonstrate that the DC is a powerful dependence measure in inferring GRNs.
(DOCX)

Table S3 **Comparison of ROC area and PR area of MIC-based algorithms and DC-based algorithms on SOS network in E.coli.data.** All of the results show that DC is significantly superior to the MIC in GRNs inference, which demonstrate that the DC is a powerful dependence measure in inferring GRNs.
(DOCX)

Supporting Information S1 **The source data and code used in this article can be freely downloaded at:** https://github.com/xiangdiuxiu/NetworkDC.
