# Supplementary material for: Inferring Nonlinear Gene Regulatory Networks from Gene Expression Data Based on Distance Correlation
Source: PLoS One. 2014 Feb 14;9(2):e87446. doi: 10.1371/journal.pone.0087446 (PMC3925093; doi:10.1371/journal.pone.0087446)
Supplement: Table S1 — Comparison of ROC area and PR area of MIC-based algorithms and DC-based algorithms on DREAM3 challenge Yeast dataset in size 10, 50, 100, respectively. All of the results show that DC is significantly superior to the MIC in GRNs inference, which demonstrate that the DC is a powerful dependence measure in inferring GRNs. (DOCX) [file pone.0087446.s006.docx]

**Table S1.** Comparison of ROC area and PR area of MIC-based algorithms and DC-based algorithms on DREAM3 challenge Yeast dataset in size 10, 50, 100, respectively.

| Method | CLR-MIC | CLR-DC | MRNET-MIC | MRNET-DC | REL-MIC | REL-DC |
| --- | --- | --- | --- | --- | --- | --- |
| ROC area |  |  |  |  |  |  |
| Size10 | 0.63 | 0.99 | 0.63 | 0.81 | 0.63 | 0.99 |
| Size50 | 0.54 | 0.89 | 0.52 | 0.76 | 0.56 | 0.89 |
| Size100 | 0.54 | 0.87 | 0.54 | 0.78 | 0.54 | 0.86 |
| PR area |  |  |  |  |  |  |
| Size10 | 0.38 | 0.94 | 0.40 | 0.97 | 0.36 | 0.92 |
| Size50 | 0.07 | 0.5 | 0.07 | 0.52 | 0.07 | 0.47 |
| Size100 | 0.02 | 0.43 | 0.04 | 0.36 | 0.04 | 0.35 |
