# Supplementary material for: Inferring Nonlinear Gene Regulatory Networks from Gene Expression Data Based on Distance Correlation
Source: PLoS One. 2014 Feb 14;9(2):e87446. doi: 10.1371/journal.pone.0087446 (PMC3925093; doi:10.1371/journal.pone.0087446)
Supplement: Table S2 — Comparison of ROC area and PR area of MIC-based algorithms and DC-based algorithms on SynTReN datasets with noise 0.1, 0.2, 0.3, respectively. All of the results show that DC is significantly superior to the MIC in GRNs inference, which demonstrate that the DC is a powerful dependence measure in inferring GRNs. (DOCX) [file pone.0087446.s007.docx]

**Table S2.** Comparison of ROC area and PR area of MIC-based algorithms and DC-based algorithms on SynTReN datasets with noise 0.1, 0.2, 0.3, respectively.

| Method | CLR-MIC | CLR-DC | MRNET-MIC | MRNET-DC | REL-MIC | REL-DC |
| --- | --- | --- | --- | --- | --- | --- |
| ROC area |  |  |  |  |  |  |
| 0.1 noise | 0.43 | 0.86 | 0.44 | 0.78 | 0.38 | 0.84 |
| 0.2 noise | 0.43 | 0.73 | 0.44 | 0.63 | 0.38 | 0.64 |
| 0.3 noise | 0.42 | 0.72 | 0.43 | 0.62 | 0.40 | 0.64 |
| PR area |  |  |  |  |  |  |
| 0.1 noise | 0.02 | 0.20 | 0.02 | 0.06 | 0.01 | 0.07 |
| 0.2 noise | 0.02 | 0.14 | 0.02 | 0.06 | 0.01 | 0.07 |
| 0.3 noise | 0.02 | 0.14 | 0.02 | 0.06 | 0.01 | 0.09 |
