# Supplementary material for: Inferring Nonlinear Gene Regulatory Networks from Gene Expression Data Based on Distance Correlation
Source: PLoS One. 2014 Feb 14;9(2):e87446. doi: 10.1371/journal.pone.0087446 (PMC3925093; doi:10.1371/journal.pone.0087446)
Supplement: Table S3 — Comparison of ROC area and PR area of MIC-based algorithms and DC-based algorithms on SOS network in E.coli.data. All of the results show that DC is significantly superior to the MIC in GRNs inference, which demonstrate that the DC is a powerful dependence measure in inferring GRNs. (DOCX) [file pone.0087446.s008.docx]

**Table S3.** Comparison of ROC area and PR area of MIC-based algorithms and DC-based algorithms on SOS network in *E.coli.*data

| Method | CLR-MIC | CLR-DC | MRNET-MIC | MRNET-DC | REL-MIC | REL-DC |
| --- | --- | --- | --- | --- | --- | --- |
| ROC area |  |  |  |  |  |  |
| SOS | 0.61 | 0.72 | 0.57 | 0.78 | 0.61 | 0.85 |
| PR area |  |  |  |  |  |  |
| SOS | 0.72 | 0.77 | 0.66 | 0.79 | 0.66 | 0.86 |
